# Nurses’ knowledge, attitudes, and practice with regards to nutritional management of diabetes mellitus

**DOI:** 10.1186/s12909-023-04178-4

**Published:** 2023-03-28

**Authors:** Mahsa Farzaei, Shahla Shahbazi, Neda Gilani, Alireza Ostadrahimi, Leila Gholizadeh

**Affiliations:** 1 Department of Medical-Surgical Nursing, Faculty of Nursing and Midwifery, Tabriz University of Medical Sciences, P.O. Box 51745347, Tabriz, Iran; 2 Department of Medical- Surgical Nursing, Faculty of Nursing and Midwifery& Clinical Research Development Unit, Sina Educational, Research and Treatment Center, Tabriz University of Medical Sciences, P.O. Box 51745347, Tabriz, Iran; 3 Department of Statistics and Epidemiology, Faculty of Health, Tabriz University of Medical Sciences, Tabriz, Postal code: 166614711 Iran; 4 Nutrition Research Center, Department of Clinical Nutrition, School of Nutrition & Food Sciences, Tabriz University of Medical Sciences, Tabriz, Postal code: 5166614711 Iran; 5 Faculty of Health, University of Technology, PO Box 123, Broadway, Sydney, NSW 2007 Australia

**Keywords:** Nurses, Knowledge, Attitudes, Practices, Nutritional, Management, Diabetes mellitus

## Abstract

**Background:**

The prevalence of diabetes is increasing rapidly worldwide. Nurses work collaboratively with multidisciplinary teams to improve diabetes management. Yet, little is known about nurses’ role in nutritional management of diabetes. This study aimed to evaluate nurses’ knowledge, attitudes, and practice (KAP) toward nutritional management of diabetes.

**Methods:**

This cross-sectional study was conducted with 160 nurses, who were recruited between July 4 and July 18, 2021 from two referral tertiary teaching hospitals in Iran. A validated paper-based self-reported questionnaire was used to assess nurses’ KAP. Data were analyzed using descriptive statistics and multiple linear regression analysis.

**Results:**

The mean knowledge score of nurses about nutritional management of diabetes was 12.16 ± 2.83, and 61.2% showing a moderate knowledge level on nutritional management of diabetes. The mean attitudes score was 60.68 ± 6.11, with 86.92% of participants demonstrating positive attitudes. The mean practice score of study participants was 44.74 ± 7.81, with 51.9% having a moderate level of practice. Higher knowledge scores were observed among male nurses (B = -7.55, *p* = 0.009) and those with blended learning as a preferred learning method (B = 7.28, *p* = 0.029). Having an opportunity to provide education to patients with diabetes during shifts affected nurses’ attitudes positively (B = -7.59, *p* = 0.017). Practice scores were higher among nurses who perceived themselves competent in the nutritional management of diabetes (B = -18.05, *p* = 0.008).

**Conclusion:**

Nurses’ knowledge and practice in the nutritional management of diabetes should be increased to help improve the quality of the dietary care and patient education they provide these patients. Further studies are needed to confirm the results of this study both in Iran and internationally.

## Background

Diabetes mellitus is one of the fastest growing chronic diseases globally, with a significant burden on individuals and societies [1]. Management of diabetes includes nutritional therapy, exercise, monitoring, pharmacologic therapy, and patient education. Nutritional management is a key element of diabetes care. An effective nutritional management helps achieve and maintain glycemic control and contributes to the well-being and quality of life of patients [2]. While registered dietitians or nutritionists take the main responsibility in nutritional assessment and management of patients with diabetes, nurses and other members of the health care team must be knowledgeable about diabetes nutrition therapy and support patients in implementation of nutritional and lifestyle changes [3].

Nurses are more likely to encounter patients with diabetes as the number of associated hospital admissions rises. Further, they are in unique position to improve the outcomes for patients with diabetes due to the length of time they spend with patients. The holistic nature of the nursing profession requires nurses to be informal nutrition advisors [4], and ensure that patients in hospital receive an appropriate diet. They assist patients at meal times, during which patients may seek dietary advice [5]. Thus, nurses should be aware of the guidelines for the nutritional management of diabetes [6].

Yet, results of previous studies suggest significant knowledge gap among nurses with regards to nutritional management of patients with diabetes [7, 8]. Poor knowledge of health care providers of dietary requirements of patients with diabetes can lead to a poor glycemic control and increase the risk of diabetes-associated complications [9]. Further, nurses had the lowest attitudes towards diabetes management among healthcare professionals in the study by Babelgaith et al. [10]. Nurses’ negative attitudes can affect their practice on nutritional management of diabetes. Other factors include time, nutrition education, organizational support, and resource availability [11–13].

Strategies to reduce the incidence of hypoglycemia and hyperglycemia are imperative to patient safety, and effective nutritional management can help achieve and maintain target glycemic control. Therefore, it is important that nurses have adequate knowledge and positive attitudes toward nutritional management in patients with diabetes to improve their practice [14, 15]. The healthcare system in Iran has recently mandated a comprehensive assessment of all patients on their hospital admission. As part of this program, nurses assess the nutritional status of patients and report those with a poor nutritional status, including those with poorly controlled diabetes, to the physician to request a nutritional consultation. Adequate knowledge, positive attitudes, and good practice of nurses are important to achievement of therapeutic goals for patients in diabetes and supporting patients in their self-care practice [15,16 ].

This study was underpinned by the KAP model, which considers using a structured standardized questionnaire to quantify and analyze what is known (knowledge), believed (attitudes), and done (practices) by a target population with regard to a topic of interest. Specific to this study, we aimed to develop an understanding of the knowledge level of nurse regarding of nutritional management of diabetes, and how the knowledge affected nurses’ attitudes and practice toward nutritional management of diabetes. Overall, studies investigating nurses’ KAP with regards to nutritional management of diabetes are limited, and to the best of our knowledge, there is no such a study in Iran. This study aimed to examine nurses’ KAP with regards to nutritional management of diabetes in Iran. Understanding nurses’ KAP about nutritional management of diabetes can generate evidence to inform the development of nutritional management programs. The specific objectives were to:

1. identify nurses’ knowledge about nutritional management of diabetes.
2. identify nurses’ attitudes toward nutritional management of diabetes.
3. identify nurses’ practice on nutritional management of diabetes.
4. identify relationships between nurses’ KAP about nutritional management of diabetes.
5. Identify multiple factors that affect nurses’ KAP about nutritional management of diabetes.

## Methods

This study applied a descriptive cross-sectional design. Participants were recruited from 35 medical and surgical wards of two teaching tertiary referral hospitals in the Northwest of Iran using random sampling method. In the first step, all eligible nurses were identified from the 35 wards and their names were written on pieces of papers and folded and put in a box. The researchers then mixed the box before drawing out the required number of sample. To be eligible for the study, participants needed to have a minimum of a bachelor’s degree in nursing and be working as a fixed term registered nurse in a medical or surgical ward for at least one month. Information from a pilot study was used to calculate the sample size in this study. The required sample size increased to 156 participants when a possible dropout rate of 20% was considered. One hundred sixty participants were recruited to the study.

There is no diabetes educator position in Iran [17]. During data collection for this study, none of the participating hospitals had a designated diabetes educator or diabetes link nurse. However, both hospitals had a nutrition unit, which was responsible for providing general nutrition care for all forms of health conditions, including nutritional consultation to patients with diabetes.

### Data collection tools

Data were collected using a self-report paper-based survey package containing four sections: The first section included questions about the socio-demographic and professional characteristics of nurses. The second section included the Nutritional Management of Diabetes Knowledge Test (NMDKT), designed and validated by Mogre et al. [5]. Its original version contains 21 questions; correct answers are scored 1, and others scored 0. Higher scores on the NMDKT represent higher level of knowledge about nutritional management of diabetes mellitus. A permission to modify and use the questionnaire for the current study was obtained from the designers (personal communication, November 3, 2019). The third section was the Nurses’ Attitudes about Nutritional Management of Diabetes Questionnaire, which developed by the researchers specifically for this study based on the WHO and the American Diabetes Association nutrition guidelines and the review of the relevant literature [2, 16, 18]. It contained 15 questions and used a five-point Likert scale, with responses ranging from strongly disagree (1) to strongly agree (5). Total scores could range from 15 to 75. The scores were then standardized between 0 to100 and categorized to three categories of high (66.6–100), moderate (33.3–66.6), and poor attitudes (0-33.3).

The final section included the Nurses’ Practice on Nutritional Management of Diabetes Questionnaire. This questionnaire was also developed by the researchers. It contained 15 questions and used a four-point Likert scale, with responses ranging from never (1) to always. Total practice scores could range from 15 to 60. The scores on practice about nutritional management of diabetes mellitus were then standardized between 0 to100 and categorized to three categories of good (66.6–100), moderate (33.3–66.6) and poor practice (0-33.3).

### Validity and reliability

The survey package was submitted to a panel of 13 experts, 4 in nutrition and 9 in nursing, for evaluation of the face and content validity. The questionnaire was revised based on the comments of the review panel and resubmitted for further evaluation. For example, question 12 was removed for a cultural reason. The panel approved all the items as appropriate, assuring good content validity. In addition, the Content Validity Ratio (CVR) and content validity index (CVI) of all questionnaires were assessed and the results supported the content validity of the used tools. The CVR and CVI for were 0.99 and 0.98, respectively. The Nurses’ Attitudes about Nutritional Management of Diabetes Questionnaire had the CVR and the CVI of 0.99 and 1.00, respectively, and the Nurses’ Practice on Nutritional Management of Diabetes Questionnaire demonstrated the CVR and the CVI of 0.99 and 1.00, respectively. Using Cronbach’s alpha to measure internal consistency, a reliability coefficient of 0.65 was attained for the NMDKT, 0.83 for the Nurses’ Attitudes on Nutritional Management of Diabetes Questionnaire, and 0.90 for the Nurses’ Practice on Nutritional Management of Diabetes Questionnaire. The questionnaire took an average 17.88 ± 9.40 min to complete.

Thus, we used the NMDKT containing 20 questions in this study. Total row scores ranged from 0 to 20. The NMDKT scores were then standardized between 0 to100 and categorized to three categories of high (66.6–100), moderate (33.3–66.6) and poor knowledge (0-33.3).

### Ethical considerations

The study received ethical approval from the Regional Research Ethics Committee of Tabriz University of Medical Sciences (Approval ID: IR.TBZMED.REC.1399.844), was carried out in accordance with the Declaration of Helsinki. Potential nurses were informed about the study and what participation would entail, and all provided informed consent before participating in the study. Permission to have access to the hospitals was obtained from hospital mangers. The survey was anonymous, and participants were ensured that the data could not be traced back to individual participants or hospitals.

### Data analysis

Data were analyzed using descriptive statistics to characterize respondents’ profiles. This included reporting mean values and standard deviations for continuous variables and frequency and percentages for categorical and ordinal variables. Relationship between KAP concepts was assessed by Pearson’s correlation coefficient, and multiple linear regression analysis was used to identify the significant associates of KAP. Analyses were conducted using the IBM SPSS for Windows, Version 24.0 statistical software package. A p-value of < 0.05 was considered statistically significant.

## Results

### Characteristics of the participants

Demographic characteristics and professional and educational background of the study nurses were summarized in Table 1. All potential participants, who were invited to participate, accepted the invitations except three nurses (acceptance rate of 98.16%). Two nurses rejected the study of due to heavy workload due and one nurse due to sickness. The mean age of participants was 30.31 ± 6.32 years; the majority were female (n = 110, 68.75%), and had a Bachelor’s degree in Nursing (n = 158, 98.8%) while the remaining (n = 2, 1.2%) had a Master’s degree in Nursing in addition to a Bachelor of Nursing degree. Above half of the participants (n = 90, 56.3%) were working in medical wards and the remaining (n = 70, 43.8%) in the surgical wards. Only small number of nurses (n = 34, 21.3%) were satisfied with nutrition education they had received during university training. The majority of nurses in this study (n = 147, 91.9%) did not have a refresher training on diabetes (Table 1).

**Table 1:** Socio-demographic characteristics and professional background of the study nurses (n = 160)

Variables	Category	Frequency (%)
Age group (in years)	< 25	25 (17.2)
	26–30	78 (53.8)
	301 − 40	30 (20.7)
	≥ 41	12 (8.3)
	Mean (SD)	30.31 (6.32)
Gender	Male	50 (31.25)
	Female	110 (68.75)
Marital status	Single	62 (39.2)
	Married	96 (60.8)
Education	Bachelor of Science	158 (98.8)
	Master of Science	2 (1.2)
Having a loved one with diabetes	Yes	79 (49.4)
	No	81 (50.6)
Relation with someone who has diabetes	Parent or sibling	51 (64.6)
	Spouse and child	9 (11.4)
	Me	1 (1.3)
	others	18 (22.8)
Hospital where they were working	Hospital 1	118 (73.8)
	Hospital 2	42 (26.3)
Ward where they were working	Surgical	70 (43.8)
	Medical	90 (56.3)
Experience as a nurse (years)	≤ 2	38 (23.8)
	3–5	53 (33.1)
	5–10	27 (16.9)
	≥ 10	42 (26.3)
Satisfied with the nutrition education received during training in university	Very unsatisfied	11 (6.9)
	Unsatisfied	58 (36.3)
	Not sure	57 (35.6)
	Satisfied	34 (21.3)
Ever had a refresher course in diabetes management	No	147 (91.9)
	Yes	13 (8.1)
Preferred learning method	classroom teaching	78 (48.8)
	self-study	12 (7.5)
	virtual route	39 (24.4)
	Blended learning	31 (19.4)
Awareness and use of Initial Nursing Assessment Sheet (INAS)	Unaware of INAS	5 (3.1)
	Aware and used sometimes	32 (20.0)
	Aware and always used	123 (76.9)
Awareness of Initial Nursing Assessment Sheet completion Guideline	Unaware	13 (8.1)
	Aware but not read it	46 (28.8)
	Aware and always used it as reference	101 (63.1)
Awareness of the National Diabetes Guideline	Yes	25 (15.8)
	No	133 (84.2)
Average number of diabetes patients cared for in a month	≤ 5	40 (26.3)
	6–10	62 (40.8)
	11–15	23 (15.1)
	≥ 16	27 (17.8)
Providing diabetes education for patients in shifts	Yes	155 (96.9)
	No	5 (3.1)
Type of diabetes education	Individual	112 (70.4)
	Group	1 (0.6)
	Individual with presence of family member	46 (28.9)
Ever counseled a diabetes patient	Yes	91 (56.9)
	No	69 (43.1)
Perceived competence in nutritional management of diabetes mellitus	I am incompetent	10 (6.3)
	I am somewhat incompetent	44 (27.5)
	I am somewhat competent	90 (56.3)
	I am competent	16 (10.0)
Desire to work as a diabetes link nurse	Yes	61 (38.1)
	No	99 (61.9)

The first objective of this study was to identify nurses’ knowledge about nutritional management of diabetes. The result showed that the mean knowledge score was 12.16 ± 2.83, with minimum score of 4 and maximum score of 18. Based on standardized scores, 93(61.2%) participants demonstrated a moderate level of knowledge (Table 2).

**Table 2:** Knowledge about nutritional management of diabetes among the study participants (n = 160)

No	Questionnaire item	Correct responses n(%)	Incorrect responses n(%)
1	Diabetes patients should not exclude any nutrient from their diet.	99 (61.9)	61 (38.1)
2	Diabetic diet is calculated based on Carbohydrates, proteins and fats	86 (54.94)	72 (45.6)
3	Trans-fats increases LDL^*^ cholesterol levels.	106 (66.3)	54 (33.8)
4	Use total carbohydrates on food labels to determine amount of carbohydrates per serving.	29 (18.6)	127 (81.4)
5	In the following breakfast, which items will raise blood sugar levels?	122 (76.7)	37 (23.3)
6	The total amount of carbohydrates is more important than the type of carbohydrate.	88 (55.0)	72 (45.0)
7	Diabetes is indicated by an FPG^**^ of 126 mg/dl or higher.	105 (65.6)	55 (34.4)
8	Symptomatic hypoglycemia could be treated using 3–4 cubes of sugar.	125 (78.1)	35 (21.9)
9	Non-fat or low fat milk contains less fat and low calories than whole milk.	63 (39.4)	97 (60.6)
10	50–60% of the daily caloric intake of diabetics should come from carbohydrates.	42 (26.3)	118 (73.8)
11	Diabetes patients should consume fruits.	152 (95.0)	8 (5.0)
12	Only carbohydrates have to be restricted for the diabetic patients.	120 (75.0)	40 (25.0)
13	Animal fat should be restricted for diabetes patients.	52 (32.5	108 (67.95)
14	Exercise plays an important role in the prevention and management of diabetes.	157 (98.1)	3 (1.9)
15	Diabetes is caused by high sugar intake.	87 (54.4)	73 (45.6)
16	Diabetes and obesity are closely related.	153 (95.6)	7 (4.4)
17	Diabetes is related to hypertension.	123 (76.9)	37 (23.1)
18	Diabetes patients should eat balanced diets.	142 (89.3)	17 (10.7)
19	10–15% of the daily caloric intake of diabetics should come from protein.	21 (13.1)	139 (86.9)
20	Cholesterol should be restricted to 300 mg daily for diabetes patients.	69 (43.1)	91 (56.9)

^*^ LDL (low-density lipoprotein); ^**^FPG (fasting plasma glucose); ^***^ Based on standardized score

The second objective was to identify nurses’ attitudes toward nutritional management of diabetes. The mean attitudes score was 60.68 ± 6.11. Based on standardized scores, 133(86.92%) participants demonstrated positive attitudes toward nutritional management of diabetes (Table 3).

**Table 3:** Attitudes toward nutritional management of diabetes among the study nurses (n = 160)

No	Attitude statements	Response, n(%)				
		1 strongly disagree	2 Disagree	3 Neutral	4 agree	5 strongly agree
1	Diet is important in controlling blood sugar for all patients with diabetes.	--	1 (6)	3 (1.9)	102 (63.8)	54 (33.8)
2	Initial nutritional evaluation is not necessary for all patients with hospitalized diabetes. *	5 (3.1)	8 (5.0)	28 (17.5)	82 (51.3)	37 (23.1)
3	Initial nutritional assessment of patients with diabetes is one of the responsibilities of nurses.	1 (0.6)	13 (8.2)	27 (17.0)	90 (56.6)	28 (17.6)
4	BMI of patients with diabetes should be calculated and interpreted at the time of admission to the ward.	4 (2.5)	5 (3.1)	21 (13.2)	100 (62.9)	29 (18.2)
5	Obese patients with diabetes are more prone to diabetes complications than normal weight patients.	--	2 (1.3)	6 (3.8)	86 (53.8)	66 (41.3)
6	All patients with diabetes should be aware of their diabetic diet.	2 (1.3)	1 (0.6)	6 (3.8)	86 (53.8)	81 (50.9)
7	Nutritional care of hospitalized patients with diabetes is the sole responsibility of the hospital’s nutritionist. *	15 (9.4)	27 (16.9)	42 (26.3)	61 (38.1)	15 (9.4)
8	All patients with diabetes should try to control their blood sugar by modifying their lifestyle to reduce complications.	1 (0.6)	3 (1.9)	4 (4.4)	82 (51.3)	67 (41.9)
9	Nutrition, diet, weight control and increased activity are the basis of diabetes control.	--	1 (0.6)	10(6.3)	78 (49.1)	70 (44.0)
10	Nurses and other members of the health care team should be aware of nutritional therapy and patient support that require nutritional and lifestyle modifications.	1 (0.6)	2 (1.3)	16 (10.0)	91 (56.9)	50 (13.3)
11	The nurse plays an important role in informing the nutritionist and patients’ understanding of the diabetic diet.	--	3 (1.9)	13 (8.1)	108 (87.5)	36 (622.5)
12	Educating patients with diabetes about the importance of a diabetic diet is one of the responsibilities of nurses.	--	7 (4.4)	23 (14.4)	98 (61.3)	32 (20.0)
13	The nurse plays an important role in strengthening the patient and family’s understanding of the diabetic diet.	1 (0.6)	4 (2.5)	16 (10.2)	104 (66.2)	32 (20.4)
14	In order to monitor the nutritional status of patients with diabetes, nurses should monitor the work of nurse assistants in helping patients with nutrition.	--	20 (12.5)	27 (16.9)	89 (655.6)	24 (15.0)
15	Nurses should evaluate the effectiveness of nutritional interventions in patients with diabetes.	1 (0.6)	2 (1.3)	13 (18.8)	91 (6.9)	36 (622.5)

*Reversed item

The third objective was to identify nurses’ practice on nutritional management of diabetes. The mean practice score was 44.74 ± 7.8. Based on standardized scores 82(51.9%) of participants demonstrated a moderate level practice (Table 4).

**Table 4:** Participants’ practice with regards to nutritional management of diabetes (n = 160)

No	In the routine care of patients with diabetes:	Never	Sometimes	Often	Always
1	I assess the nutritional needs of patients with diabetes using the nurse initial evaluation sheet.	--	19 (11.9%)	75(46.9%)	66(41.3%)
2	I calculate and interpret the body mass index (BMI) of a patient with diabetes.	8 (5.0%)	25 (15.6%)	67 (41.9%)	60 (37.5%)
3	During the initial assessment of the patient, I ask him or her companion about recent weight loss or gain	1(0.6%)	21 (13.1%)	70 (43.8%)	68 (42.5%)
4	I make nursing diagnoses related to the nutrition of patients with diabetes and record them in the nurse report sheet for follow-up.	2(1.3%)	37 (23.1%)	75 (46.9%)	46 (28.8%)
5	I prepare and adjust a nursing care plan for each patient with diabetes based on primary and secondary information.	19 (11.9%)	19 (11.9%)	19 (11.9%)	19 (11.9%)
6	In the ward, I monitor that the type and amount of food required by patients with diabetes is in accordance with the diet set by the nutrition consultant, and if the patient wishes to change the type or amount of food, I coordinate with the nutritionist.	9 (5.6%)	51 (31.9%)	74 (46.3%)	26 (16.3%)
7	Based on the results of the patient’s initial assessment, I will inform the treating physician that the patient has diabetes in order to seek nutritional advice.	28 (17.5%)	42 (26.3%)	61 (38.1%)	29 (18.1%)
8	I follow up on informing the nutritionist about the patient’s nutritional status and conducting nutrition counseling.	24(15.0%)	47 (29.4%)	60 (37.5%)	29 (18.1%)
9	During the rounds / visits, I discuss the nutritional status of my diabetic patients.	7 (4.4%)	44 (27.5%)	75 (46.9%)	34 (21.3%)
10	In order to strengthen the understanding of patients with diabetes and their families, I teach them about the diabetic diet.	1 (0.6%)	23 (14.5%)	78 (49.1%)	57 (35.8%)
11	I evaluate the effectiveness of nutritional training provided to patients with diabetes in various ways, including the Teach back method, test results, and so on.	5 (3.1%)	43 (26.9%)	69 (43.1%)	43 (26.9%)
12	I monitor the nutritional and nutritional needs of patients with diabetes in a variety of ways (for example, after insulin injections / oral antidiabetic medications, I visit the patient to make sure he or she has eaten the food).	--	24 (15.0%)	89 (55.6%)	47 (29.4%)
13	At the time of discharge of a patient with diabetes, I provide oral instruction to patients / their families on nutrition and diabetic diet.	1(0.6%)	23 (14.5%)	78 (49.1%)	57 (35.8%)
14	At the time of discharge of a patient with diabetes, I provide written information to patients / their families about nutrition and diabetic diet.	5(3.1%)	50 (31.3%)	61 (38.1%)	44 (27.5%)
15	I record the discharge training provided for the diabetic diet in the patient education form.	3(1.9%)	20 (12.5%)	79 (49.4%)	58 (36.3%)

The forth objective of this study was to identify relationships between nurses’ KAP about nutritional management of diabetes. The correlation between knowledge, attitudes, and practice was evaluated using the Pearson’s correlation analysis. There was a statistically significant correlation between both knowledge (r= -0.164, *p* = 0.045) and attitudes (r = 0.361, *p* < 0.001) with practice scores. However, the correlation between knowledge and attitudes was not statistically significant (r=-0.067, *p* = 0.423) (Table 5).

**Table 5:** Correlation between the knowledge, attitudes, and practice scores of the study nurses with regards to nutritional management of diabetes (n = 160)

Variable	knowledge		Attitude		Practice	
	r*	p-Value	r*	p-Value	r*	p-Value
**Knowledge**	1	--				
**Attitude**	-0.067	0. 423	1	--		
**Practice**	-0.164	0.045	0.361	< 0.001	1	--

* Pearson correlation coefficient

In the multiple linear regression analysis, gender and preferred method of learning were statistically significant correlates of participants’ knowledge of nutritional management of diabetes (Table 6). Higher knowledge was reported among male nurses (B = -7.55, *p* = 0.009), and those reported blended learning as their preferred learning method (B = 7.28, *p* = 0.029).

**Table 6:** Relationship between the knowledge scores and the sociodemographic characteristics and professional background of the study nurses (n = 160)

Variables	Categories	Multiple regression	
		β (95% CI(	p-Value
Gender	Female	-7.55 (-13.19 to -1.91)	0.009
	Male	reference category	
Age group (years)	< 25	5.66 (-9.23 to 20.54)	0.453
	26–30	9.20 (-3.70 to 22.09)	0.161
	31–40	7.57 (-2.18 to 17.32)	0.127
	> 41	reference category	
Marital status	single	-4.27 (-9.99 to 1.45)	0.142
	married	reference category	0.559
Hospital where employed	Hospital 1	1.64 (-3.91 to 7.19)	0.559
	Hospital 2	reference category	
Nursing experience(years)	< 2	-7.55 (-19.27 to 8.18)	0.205
	3–5	-5.94 (-17.27 to 5.39)	0.301
	5–10	-2.66 (-12.50 to 7.19)	0.594
	> 10	reference category	
Satisfied with the nutrition education received during training in university	Very unsatisfied	5.74 (-3.72 to 15.21)	0.232
	Unsatisfied	5.27 (-1.27 to 11.80)	0.113
	Not sure	1.77 (-4.61 to 8.15)	0.584
	Satisfied	reference category	
Preferred *learning* method	Face to face	7.28 (0.75 to 13.80)	0.029
	self-study	7.78 (-2.59 to 18.15)	0.140
	virtual route	5.57 (-1.72 to 12.80)	0.133
	Blended learning	reference category	
Awareness and use of Initial Nursing Assessment Sheet (INAS)	Unaware of INAS	9.45 (-3.17 to 22.07)	0.141
	Aware and used sometimes	-0.87 (-7.27 to 5.52)	0.787
	Aware and always used	reference category	
Awareness of National Diabetes document	Yes	6.26 (-6.68 to 7.20)	0.940
	No	reference category	
Providing diabetes education	Yes	-7.17 (-2.0 to 5.99)	0.283
	No	reference category	

Only provision of diabetes education during working shifts was the statistically significant correlate of attitudes toward nutritional management of diabetes (Table 7). Positive attitudes scores were reported by nurses who provided diabetes education for patients during their work shifts (B = -7.59, *p* = 0.017).

**Table 7:** Relationship between the attitude scores and the sociodemographic characteristics and professional background of the study participants (n = 160)

Variables	Categories	Multiple regression	
		β (95% CI(	p-Value
Relation with someone who have diabetes	Parent or sibling with diabetes	-1.86 (-8.16 to 4.43)	0.555
	Spouse and child	-1.185 (-10.71 to 7.01)	0.678
	Myself	15.08 (-7.03 to 37.18)	0.177
	Others	reference category	
*Awareness of* Initial Nursing Assessment Sheet completion Guideline	Unaware	-1.49 (-12.37 to 9.38)	0.784
	Aware but not read it	1.79 (-7.06 to 3.47)	0.498
	Aware and always used it as reference	reference category	
Awareness of National Diabetes document	Yes	4.98 (-1.18 to 11.15)	0.111
	No	reference category	
No of diabetes patient during one months	≤ 5	-4.90 (-13.56 to 3.75)	0.261
	6–10	1.50 (-7.52 to 10.15)	0.740
	11–15	-2.12 (-12.63 to 8.38)	0.686
	≥ 16	reference category	
Type of diabetes education	Individual	-3.57 (-9.05 to 1.90)	0.197
	Group	11.47 (-7.51 to 30.45)	0.231
	Individual with presence of family member	reference category	
Providing diabetes education for patients during shifts	Yes	-7.59 (-13.80 to -1.38)	0.017
	No	reference category	
Ever counselled a diabetes patient	Yes	1.93 (-3.26 to 7.12)	0.459
	No	reference category	
Perceived competence in nutritional management of diabetes mellitus	I am incompetent	0.745 (-11.05 to 12.54)	0.900
	I am somewhat incompetent	1.93 (-7.12 to 10.99)	0.670
	I am somewhat competent	− 0.463 (-9.22 to 8.29)	0.916
	I am competent	reference category	
Desire to assume diabetes link nurse role	Yes	2.83 (-2.64 to 8.30)	0.304
	No	reference category	

The multiple linear regression analysis showed statistically significant relationships between hospital and feeling competent and practice on nutritional management of diabetes (Table 8). Higher practice scores were observed among nurses who were employed in hospital 2 and those who perceived themselves competent in the nutritional management of diabetes (B = -18.05, *p* = 0.008).

**Table 8:** Relationship between the practice scores and the professional background of study participants (n = 160)

Variables	Categories	Multiple regression	
		β (95% CI(	p-Value
Hospital where employed	Hospital 1	-8.52 (-14.57 to -2.47)	0.006
	Hospital 2	reference category	
Have you had any refresher course in nutritional management of diabetes	No	-7.63 (-17.36 to 2.10)	0.124
	Yes	reference category	
Perceived competence in nutritional management of diabetes mellitus	I am not competent	-18.05 (-31.35 to -4.75)	0.008
	I am somewhat not competent	-10.42 (-20.14 to -0.70)	0.036
	I am somewhat competent	-9.43 (-18.39 to -0.48)	0.039
	I am competent	reference category	

## Discussion

The findings of this study provide insights into nurses’ KAP about the nutritional management of diabetes. Overall, participants demonstrated a moderate level of knowledge on the nutritional management of diabetes. Knowledge forms the basis of professional practice. Knowledge deficits of diabetes care, including nutritional management of diabetes, imposes a significant risk to delivery of safe practice [7]. The results of a review study revealed a significant knowledge deficit in the core aspects of diabetes care among nurses globally [7]. Comparing our findings with the past research, the mean knowledge score in our study was higher than the overall 44% correct responses reported by Mogre et al. [5]. Also, Naz et al. reported that half nurses in their study had an unsatisfactory level of knowledge about diabetes and diabetes meal planning [6]. Badshah et al. found that the majority of nurses had poor knowledge regarding diabetic diet [19]. Likewise, the study by Oyewole et al. revealed nurses’ knowledge deficiency in some critical areas, such as diabetes diet [20]. Inadequate nutritional knowledge of nurses could lead to inaccurate information provided to diabetic patients, which may lead to poor diabetes management and an increase in the rate of diabetes-related complications and treatment costs [19].

The moderate level of knowledge found among the nurses in our study could be due the fact that nurses mostly had not received any diabetes education after graduation from university. Similar to the present study results, Samancioglu et al. reported that only 3.9% of the nurses had a certificate as a ‘diabetes educator’ in Turkey [21]. Likewise, Alhaiti et al. reported that most nurses in Saudi Arabia in their study (78.4%) had not received any refreshing training on diabetes [22]. Like many other countries [23], nurses in Iran receive 26 h of education on nutrition during their bachelor of nursing degree. Considering the dramatic rise in the prevalence of diabetes worldwide, educational curriculums in health-related fields should better focus on training health care professionals about diabetes care, including the nutritional management of diabetes [24].

The majority of nurses in our study demonstrated positive attitudes toward the nutritional management of diabetes mellitus, which is a promising finding. Similar to the present study, a study by Kim and Choue showed that most Korean nurses possessed positive attitudes about attending to nutritional needs of patients and showed a high desire to receive further training on patient nutrition [14]. In contrast to the current study, Oyewole et al. in Nigeria reported that 48.9% of nurses in their study exhibited negative attitudes toward diabetes care in general [20]. Positive attitudes can be considered as an opportunity to improve the knowledge and practice of nurses on the nutritional management of diabetes mellitus.

Overall nurses in the current study reported a moderate level of practice on the nutritional management of diabetes. Direct comparison to other studies is difficult due to limited studies assessing nurses’ practice with regards to nutritional management of diabetes. The available studies mostly assessed nurses’ practice in relation to diabetes in general or addressed nutritional management in hospitalized patients in general, with a few questions targeted on diabetes. Comparing our findings with available evidence, the mean practice score achieved by the nurses in this study was higher than scores reported by Emami et al. in Iran [18]. In the Emami et al.’s study, nurses acted poorly on nutritional screening and the subsequent referral to a dietician for professional assessment. Initial nutritional assessment of chronically ill patients, including those with poorly controlled diabetes can have a significant effect on patient outcomes. A reason for the suboptimal practice of nurses in our study could be due to time constraint, making the process of initial nutritional assessment of patients and their referral to a dietitian unrealistic in the clinical settings.

The current study found a positive correlation between knowledge and attitudes with practice. The research team could not find any study that attempted to find the correlation between KAP about nutrition management of diabetes among nurses in Iran. The knowledge scores of nurses about nutritional management of diabetes were significantly higher among male nurses and nurses who selected blended learning as their preferred learning method. Similar to this finding, in the study conducted by Mogre et al. in Ghana, male nurses scored higher than female nurses on the NMDKT [5].

Furthermore, the present study revealed an association between attitude scores and involvement in diabetes education. Nurses who provided education to diabetic patients during their work possessed more positive attitudes toward nutritional management of diabetes. Higher practice scores were observed among nurses who were employed in hospital 2. One reasonable explanation to this finding could be that hospital 1 had an endocrine ward, admitting most patients with diabetes. This might have caused the nurses of other wards in hospital 1 have less exposure to patients with diabetes, and develop competency in managing the nutritional care needs of patients with diabetes. Supporting this explanation, practice scores in this study were positively associated with nurses’ perceived competency in nutritional management of diabetes.

## Limitations

The results of this study contributed to our understanding of nurses’ KAP on the nutritional management of diabetes in Iran. Using random sampling method and recruiting participants from two tertiary referral teaching hospitals increase the generalizability of the findings. However the self-report nature of the data is a limitation. Also, this study was conducted in hospital settings, it should be noted that only small percentage of patients with diabetes are admitted to hospitals, and the main part of diabetes care is provided in primary care settings or outpatient clinics. Thus, the results of this study are applicable to hospital settings only.

## Conclusions

Nurses in this study demonstrated a moderate level of knowledge and practice in relation to the nutritional management of diabetes mellitus, although their attitude toward this aspect of patient care was positive. Being a male nurse, having a preference for blended learning, having opportunities to get involved in providing education to diabetes patients during work, hospital where employed, and perceive competency in the nutritional management of diabetes affected nurses’ knowledge, attitudes, and practice. Given the growing trend of diabetes worldwide and the role of nutrition in diabetes management, it is necessary to improve the knowledge, attitudes, and practice of nurses about the nutritional management of diabetes. The educational curriculums should be examined for adequate education of nurses about diabetes care.

## Data Availability

The datasets generated and/or analyzed during the current study are not publicly available due to agreements with participants who restricted data sharing but are available from the corresponding author on reasonable request.
